# Retention Mechanism Studies of Selected Amino Acids and Vitamin B6 on HILIC Columns with Evaporative Light Scattering Detection

**DOI:** 10.1007/s10337-013-2502-y

**Published:** 2013-07-10

**Authors:** Sylwia Noga, Pavel Jandera, Bogusław Buszewski

**Affiliations:** 1 Chair of Environmental Chemistry & Bioanalytics, Faculty of Chemistry, Nicolaus Copernicus University, 7 Gagarin St., 87-100 Toruń, Poland; 2 Department of Analytical Chemistry, Faculty of Chemical Technology, University of Pardubice, 53210 Pardubice, Czech Republic

**Keywords:** Hydrophilic interaction liquid chromatography, Evaporative light scattering detection, Retention mechanism, Amino acids, Vitamin B6

## Abstract

The goal of the study was to investigate separation mechanism of selected “essential” amino acids (leucine, isoleucine, threonine, tryptophan, proline, and glycine) and vitamin B6 in hydrophilic interaction liquid chromatography (HILIC) with the evaporative light scattering detection. Chromatographic measurements were made on three different HILIC columns: amide-silica (TSK-gel Amide-80), amino-silica (TSK-gel NH_2_-100), and cross-linked diol (Luna HILIC). The retention behaviour of the analytes was investigated as a function of different binary hydro-organic mobile phases containing 10–90 % (v/v) acetonitrile. The compounds studied were separated under isocratic and gradient conditions. The best results of tested biologically active compounds separation were obtained on the TSK-gel NH_2_-100 column. TSK-gel NH_2_ column showed mixed HILIC–ion-exchange mechanism, the highest separation efficiency and better selectivity and resolution for tested analytes than the other studied column, especially at concentration of water in mobile phase lower than 30 % (v/v*)*. Special attention was dedicated to the study of interactions among the stationary phase, mobile phase and the analytes.

## Introduction

Amino acids are very important compounds from a biological point of view. They perform biological roles including neurotransmitters, transport, and in synthesis. 9 of the 20 standard amino acids are called “essential” amino acids for humans, because they cannot be synthesized de novo by the organism, therefore must be supplied in the diet. The amino acids regarded as essential for humans are phenylalanine, valine, threonine, tryptophan, isoleucine, methionine, leucine, lysine, and histidine. In addition, the amino acids like glycine or proline are considered conditionally essential, meaning they are not normally required in the diet, but must be supplied exogenously to specific populations that do not synthesize them in adequate amounts. Because of their biological significance, amino acids are important in nutrition and are commonly used in nutritional supplements and food technology [1, 2].

Another nutritional supplement is vitamin B6. This compound is a water-soluble vitamin and is essential in the diet. Its active form is a cofactor in many reactions of amino acid metabolism. It is also needed for the production of neurotransmitters, and proper functioning of the nervous system and the immune system. Vitamin B6 is widely distributed in foods in both its free and bound forms [3–5].

The high polarity of biologically active compounds, their low volatility and their lack of a strong chromophore group make their separation and detection difficult. In order to solve this problem, derivatization approaches were developed with the purpose of increasing analyte volatility (for GC-flame ionization detection/MS analysis) or creating amino acid derivatives with strong chromophore/fluorophore groups (for LC or CE-UV/fluorescence analysis). However, all the existing derivatization methods present one or more of the following drawbacks: derivative instability, reagent interferences, long preparation time, inability to derivatize the secondary amino groups, increased void volume for the post-column derivatization methods, long chromatographic separation of certain amino acids derivatives, and problems with derivatization towards specific amino acids [6–12]. An alternative to derivatization methods is direct amino acid detection. It has been achieved by the evaporative light scattering detector (ELSD), the corona charged aerosol detector (cCAD), and the electrospray mass spectrometer (ESI–MS) [13–22].

The ELSD detector has been commercially available for many years [23–25]. A general detection mechanism comprises three stages: nebulization, mobile phase evaporation and detection. The ELSD is usually chosen as an alternate detector when the compounds of interest lack a UV chromophore and gradient compatibility is needed [26]. This type of detector can detect all solutes that are less volatile than mobile phase. Amino acids and vitamin B6 are non-volatile compounds and are, therefore, suitable analytes for this type of detection.

Hydrophilic interaction liquid chromatography (HILIC) was first suggested by Alpert in 1990 [27], and in recent years there has been increased interest in HILIC among scientists. The HILIC stationary phase is a polar stationary phase, such as a silica, diol, amino or amide phase and the mobile phase is composed of polar solvents, typically of acetonitrile and water, with a greater percentage of the organic solvent [30]. In HILIC mode, the separation mechanism is based on the differential distribution of the injected analyte molecules solute between the acetonitrile-rich mobile phase and a water-enriched layer adsorbed onto the hydrophilic stationary phase [27–29]. Previously published theory was mostly assuming that HILIC retention is caused by the partitioning. It is commonly accepted now that retention mechanism is a mixed mode, and the partition mechanism originally envisaged may occur together with adsorption, ion exchange, and even hydrophobic interactions under the appropriate experimental conditions [31]. The complexity of this mechanism can manifest itself in different selectivity of different HILIC phases.

The ELSD detector in the HILIC mode can produce greater sensitivity than in the reversed-phase mode, as the HILIC mobile phases are primarily organic solvent and more easily evaporated than more aqueous reversed-phase mode mobile phases.

In this work, HILIC mode was used with ELSD detection, to separate several selected “essential” amino acids (leucine, isoleucine, threonine, tryptophan, proline, and glycine) and vitamin B6. For this purpose, three typical HILIC stationary phases: amide-silica (TSK-Gel Amide-80), amino-silica (TSK-Gel NH_2_-100), and cross-linked diol (Luna HILIC) were studied. Special attention was dedicated to the study of interactions among the stationary phase, mobile phase and the analytes. Another goal was selecting the best column for separation of the tested biologically active compounds.

## Experimental

### Chemicals

**Fig. 1:**
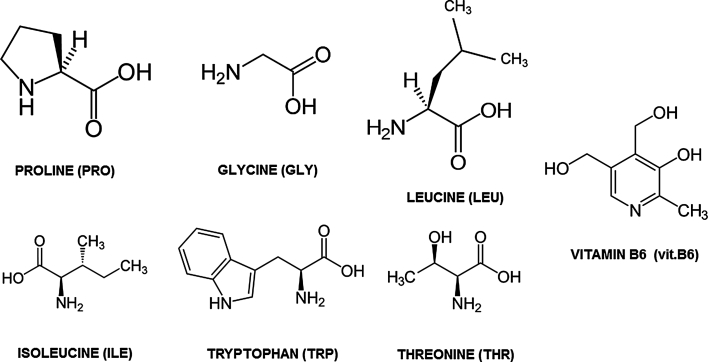
Schematic structures of the analytes

Amino acids and vitamin B6 standards were from Reanal (Budapest, Hungary). Acetonitrile (ACN) (HPLC Grade) was from Sigma-Aldrich (Steinheim, Germany). Formic acid (Merck, Darmstadt, Germany) was used as the mobile phase additive. Water was purified with a Milli-Q Water Purification System (Millipore Corporation, Bedford, MA, USA). The chemical structures of the analytes are presented in Fig. 1. The properties of tested biologically active compounds are summarized in Table 1.

**Table 1:** Properties of tested biologically active compounds from molecular modelling

No.	Compound	Abbreviated name	Molecular mass (g/mol)	log *P*	log *D* (pH 3.0)	p*K*_a_ values		p*I*
						p*K*_1_ (–COOH)	p*K*_2_ (–NH_3_^+^)	
1	Proline	PRO	115.13	−0.57	−3.15	2.35	10.70	6.48
2	Isoleucine	ILE	131.17	0.73	−1.87	2.55	9.79	6.02
3	Glycine	GLY	75.07	−1.03	−3.60	2.43	9.64	5.97
4	Leucine	LEU	131.18	0.73	−1.86	2.55	9.79	5.98
5	Threonine	THR	119.12	−1.23	−3.78	2.19	9.64	5.87
6	Tryptophan	TRP	204.23	1.04	−1.52	2.30	9.51	5.89
7	Vitamin B6	Vit.B6	169.18	−1.10	−3.03	8.96		–

### Equipments

Retention times of the test compounds were investigated using a HPLC chromatograph consisting of an LC-20AD XR pump (Shimadzu, Tokyo, Japan) and a manual injection valve (TSP, Riviera Beach, FL, USA). LCtalk HPLC version 2.03.02 software (TSP, Riviera Beach, FL, USA) was used for data collection and instrument control. The ELSD detector was model Sedex 75 from Sedere (Alfortville, France).

The structural descriptors of tested analytes were derived with the use of the HyperChem 7.0 program (HyperCube, Waterloo, Canada) and ACD/LABs (Advanced Chemistry Development Inc., Toronto, Canada). They are presented in Table 1 and were obtained after the modeling of molecules structures. The semi-empirical quantum-chemical AM1 method was used for geometry optimization.

Chromatographic measurements were performed on three different polar stationary phases with specific functionalities. The structures of bonded ligands and properties of all the columns used in the study are summarized in Table 2.

**Table 2:** Characteristics of columns used in the investigations

Column	Structure of stationary phase	Dimension (mm)	Silica particle size (μm)	Pore diameter (Å)	Surface area (m^2^ g^−1^)	Manufacturer
TSK-gel Amide-80		4.6 × 150	3	80	450	Tosoh Bioscience Gmbh, Stuttgart, Germany
TSK-gel NH_2_-100		4.6 × 150	3	100	450	Tosoh Bioscience Gmbh, Stuttgart, Germany
Luna HILIC		4.6 × 150	5	200	200	Phenomenex, Torrance, CA, USA

### Chromatographic Conditions

The HPLC analyses were carried out at room temperature (20 ± 1 °C) under isocratic and gradient conditions. The retention behaviour of the analytes was investigated as a function of mobile phase composition ranging from 10 to 90 % (v/v) water in acetonitrile. Mixtures of acetonitrile and water with formic acid (adjusted to pH 3.0) were used as the mobile phases. Before use, all mobile phases were degassed by ultrasonication. Sample solutions of amino acids and vitamin B6 were prepared in the mobile phase at concentrations yielding adequate detector response (mix stock solution with 40 μg mL^−1^ for each compound). Sample volumes of 15 μL were injected and the flow rate of the mobile phase was 1.0 mL min^−1^. The ELSD detection conditions were set as follows: the drying gas flow rate was 1.0 L min^−1^, the drift tube temperature was 40 °C, nebulizer gas pressure was 2.3 bar and the gain was set at 1.

## Results and Discussion

The retention phenomenon in HPLC depends on various types of intermolecular interactions between the solute and the stationary phase, the solute and the mobile phase, the stationary and mobile phases. In this work, we have investigated in particular the retention of six amino acids and vitamin B6 on three physic-chemically diversified stationary phases with specific functionalities (TSK-gel Amide, TSK-gel NH_2_ and Luna HILIC). The studied columns have different physicochemical properties; therefore it was interesting to compare them in HILIC systems. The experimental conditions were chosen on the basis of earlier studies. The choice of acidic pH (pH = 3.0) was made mainly with regard to the stationary phase type: a pH value of 3.0 causes protonation of some packing material functional groups.

### Choice of ELSD Conditions

Like many compounds, amino acids lack a suitable chromophore, which is necessary for UV detection. For this reason, we decided to focus our attention on the evaporative light scattering detector. This method gave an optimal response under a variety of experimental conditions, with signal-to-noise ratios always larger than UV detection. The individual parameters which could influence the response of ELSD are as follows: drift tube temperature and nebulizer gas pressure.

Using 15 μL injections of 40 μg mL^−1^ samples, the ELSD was optimized for temperature by decreasing the drift tube setting from 50 to 30 °C in increments of 5 °C, while keeping constant the mobile phase flow rate (1.0 mL min^−1^) and the nitrogen gas flow rate (1.0 L min^−1^). The S/N for all amino acids and vitamin B6 was evaluated at each interval, yielding a detector temperature of 40 °C for the highest signal with the lowest baseline noise. Next, the nitrogen gas flow rate of the detector was optimized while keeping the drift tube temperature constant at 40 °C. The nebulizer gas pressure was decreased from 4.0 to 2.0 bar, and the S/N for amino acids and vitamin B6 was evaluated at each interval. The highest signal with the least baseline noise occurred with a nebulizer gas pressure of 2.3 bar.

### Retention Studies

**Fig. 2:**
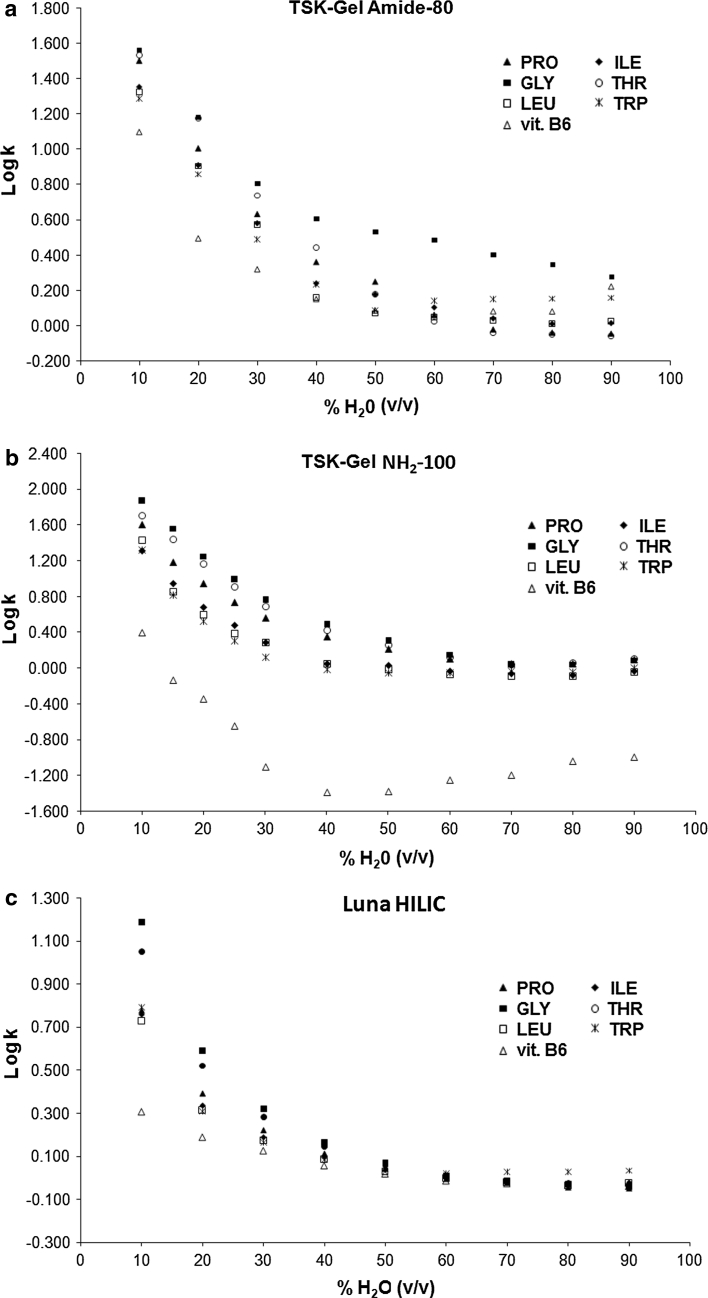
Influence of water percentage on the retention on **a** TSK-gel Amide, **b** TSK-gel NH_2_, **c** Luna HILIC. Mobile phase: acetonitrile and water with formic acid (pH 3.0) [(*x*:100 − *x*), v/v]. Flow rate: 1.0 mL min^−1^. Compounds: *PRO* proline, *ILE* isoleucine, *GLY* glycine, *THR* threonine, *LEU* leucine, *TRP* tryptophan, *vit.B6* vitamin B6

The retention behaviour of the analytes was investigated under isocratic conditions as a function of the composition of binary hydro-organic mobile phases. In the present study, only ACN was investigated as the organic modifier of the mobile phase, as it is the solvent of choice for HILIC, whatever the nature of the stationary phase. The effect of water concentration on the retention data of seven tested analytes was studied in a broad range, 10–90 % (v/v) for all columns. It is shown in Fig. 2.

For tested columns, all the retention factors decrease as the water content of the mobile phase increases. At increasing concentration of acetonitrile, water adsorbs more strongly on the surface of the polar stationary phase. The more hydrophilic are the analytes, the more the partitioning equilibrium is shifted towards the adsorbed water layer on the stationary phase, and, thus the more the analytes are retained.

For TSK-gel Amide column (Fig. 2a), the retention factors decrease with increasing concentration of water up to 40–50 % (v/v) in the mobile phase and then increases again at increasing water concentration and the analytes elute in order of decreasing polarities at high water concentrations (Table 1).

On the TSK-gel NH_2_ column, log *k* values decrease with the increase of water content in the mobile phase up to 40 % (v/v) (Fig. 2b). However, amino acids do interact more strongly with the amino-silica stationary phase than vitamin B6, when the mobile phase contains high level of organic solvent. The acid–base character of amino acids can explain this behaviour. Amino acids can behave either as weak acids or bases depending on their isoelectric points (Table 1) and solution pH. At the isoelectric point, both functional groups are charged but the molecule as a whole carries no net charge. Above a characteristic p*K*_2_ value, they are negatively charged, and below p*K*_1_, they are positively charged. Decreasing pH (from 6.0 to 3.0) extends the positive charge in acidic amino acids and reduces the negative charge in basic ones. It is known that isoelectric points of the studied amino acids are around 6.0 in aqueous solutions (Table 1). The pH of the mobile phase in present study was adjusted to 3.0 with formic acid, so that amino acids dissolved in the mobile phase contain partial positive charge located on the amino groups. However, in spite of this partial positive charge, bonded amino phases are weak anion exchangers at pH = 3, so that the positively charged bonded amino groups in the stationary phase attract the carboxylic groups of the amino acids by electrostatic interactions. These electrostatic interactions explain increased retention of amino acids on the TSK-gel NH_2_ column with respect to the other two columns, which do not contain ionizable functional groups. This behaviour is observed even in mobile phases containing more than 40 % (v/v) water. There are non-significant differences between the retention of the individual amino acids, demonstrating thus stronger ion-exchange interactions in comparison with non-polar interactions. Very low retention of vitamin B6 in this mobile phase range can be explained by repulsive electrostatic interactions of the weak anion exchanger with the positively charged secondary amino group of this compound, which does not contain ionizable carboxylic or other anionic group(s) (Fig. 2b).

On the Luna HILIC column (Fig. 2c), the retention is very low in the mobile phases containing more than 40 % (v/v) water, but the log *k* values increase rapidly with the decrease of water content in mobile phase from 30 to 10 % (v/v). It is probably due to strong hydrophilic interactions between analytes and cross-linked DIOL ligands bonded on surface of Luna HILIC. The chemically bonded DIOL phase demonstrates high polarity and does not contain ionizable groups other than non-reacted residual silanols [4]. Proline, threonine and glycine are most polar (see Table 1) and are eluted later than the other compounds. Moreover, the retention factors of amino acids and vitamin B6 are approximately constant as the water content in mobile phase increases from 50 to 90 % (v/v) (Fig. 2c). Analytes were eluted at the hold-up volume in this water concentration range, where the interactions between selected biologically active compounds and stationary phase are weak.

**Fig. 3:**
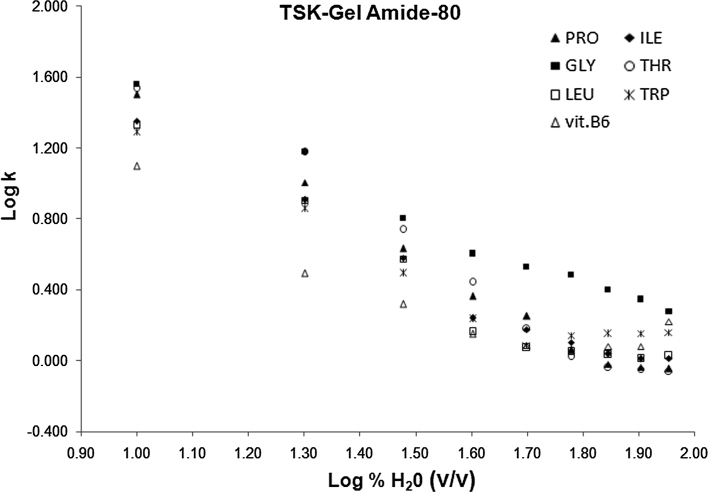
Plot of log *k* versus log (% water) considering seven model compounds, for the TSK-gel Amide column

However, to evaluate the impact of mobile phase composition on retention and selectivity not only log *k* = *f*(% water) plots were considered. As suggested by Hemström and Irgum [29], log *k* versus log (% water) was also reported (data are shown only for TSK-gel Amide-80 column) (Fig. 3).

In theory, linearity of log *k* = *f* (% water) would indicate a predominance of a partitioning process, whereas linearity of log *k* = *f* (log  % water) would reveal an adsorption mechanism. As shown in Figs. 2 and 3, in both the cases, no linearity was observed, suggesting that the mechanism is a mixture of partitioning (mostly driven by hydrophobicity) and adsorption (mainly driven by hydrogen-bonding capability), according to the model of Soczewinski–Snyder. Thus, surface adsorption via polar interaction coexisted with a partitioning mechanism with the water-enriched layer. This conclusion is in agreement with Hemström and Irgum [29], who stated that the mechanism is a mixture of partitioning and adsorption in different proportions, depending on the nature of the stationary phase, the nature of the analytes and the eluent composition.

### Separation of Amino Acids and Vitamin B6 in HILIC Mode

All the studied stationary phases were used for separation of selected biologically active compounds. The selectivity and resolution parameters listed in Table 3 give clues for the selection of the separation conditions.

**Table 3:** Separation characteristic parameters for seven-compound mixture analysis under isocratic elution conditions with 30 % (v/v) and 20 % (v/v) water in mobile phase (pH = 3.0)

No.	Compounds	Retention time *t*_R_		Retention factor *k*		Bandwiths		Selectivity (*α*)		Resolution for adjacent peaks *R*_s_		Number of theoretical plates	
		30 %	20 %	30 %	20 %	30 %	20 %	30 %	20 %	30 %	20 %	30 %	20 %
TSK-gel Amide-80													
1	Vitamin B6	3.213	4.554	2.089	3.125	0.327	0.407	–	–	–	–	535	694
2	Tryptophan	4.267	9.073	3.103	7.218	0.267	0.533	1.49	2.31	2.34	4.99	1,415	1,605
3	Leucine	4.947	9.980	3.757	8.039	0.273	0.487	1.21	1.11	1.46	1.06	1,819	2,327
4	Isoleucine	5.000	10.060	3.807	8.112	0.320	0.500	1.01	1.01	0.07	0.10	1,353	2,243
5	Proline	5.513	12.287	4.301	10.129	0.227	0.567	1.13	1.25	1.33	2.32	3,268	2,602
6	Threonine	6.733	17.653	5.474	14.990	0.340	0.627	1.27	1.48	2.09	5.04	2,173	4,391
7	Glycine	7.667	17.787	6.372	15.111	0.440	0.627	1.16	1.01	1.22	0.16	1,682	4,458
Luna HILIC													
1	Vitamin B6	2.433	2.813	1.339	1.548	0.153	0.153	–	–	–	–	1,401	1,873
2	Tryptophan	2.560	3.353	1.461	2.037	0.133	0.140	1.09	1.32	0.56	2.29	2,053	3,178
3	Leucine	2.587	3.373	1.488	2.055	0.133	0.140	1.02	1.01	0.13	0.09	2,096	3,216
4	Isoleucine	2.647	3.500	1.545	2.170	0.147	0.180	1.04	1.06	0.25	0.44	1,796	2,095
5	Proline	2.773	3.827	1.666	2.466	0.140	0.153	1.08	1.14	0.54	1.29	2,173	3,466
6	Threonine	3.040	4.767	1.923	3.318	0.153	0.167	1.15	1.34	1.00	3.27	2,187	4,514
7	Glycine	3.220	5.407	2.096	3.898	0.147	0.160	1.09	1.17	0.72	2.30	2,658	6,327
TSK-gel NH_2_-100													
1	Vitamin B6	1.560	2.207	0.780	1.456	0.173	0.193	–	–	–	–	450	724
2	Tryptophan	3.353	6.600	1.318	3.353	0.167	0.267	1.69	2.30	2.74	6.33	2,233	3,385
3	Leucine	4.253	7.473	1.941	3.941	0.220	0.273	1.47	1.18	2.40	1.96	2,070	4,151
4	Isoleucine	4.253	8.767	1.941	4.783	0.207	0.320	1.00	1.21	0.00	2.31	2,339	4,158
5	Proline	6.680	14.827	3.619	8.780	0.193	0.280	1.86	1.84	7.38	12.77	6,637	5,535
6	Threonine	8.540	23.727	4.906	14.651	0.280	0.473	1.36	1.67	3.95	11.09	5,154	6,940
7	Glycine	9.747	28.533	5.741	17.821	0.320	0.480	1.17	1.22	2.22	5.97	5,140	9,576

Selectivity (*α*) is one of the most important chromatographic parameters used for the examination and determination of specific and nonspecific interactions in various chromatographic systems. This parameter is most often presented as the ratio between the retention factor (*k*) of two substances analyzed. In Table 3, we show somewhat diverse selectivity for TSK-gel amide, Luna HILIC and TSK-gel NH_2_ columns for the separation of a mixture of tested analytes. The differences in selectivity take place among the various columns when using the identical mobile phase. The best selectivity has been obtained for the TSK-gel NH_2_ column; the selectivity on the TSK-gel Amide and Luna HILIC is too low and these columns are not suitable for the separation of the amino acids tested and vitamin B6.

**Fig. 4:**
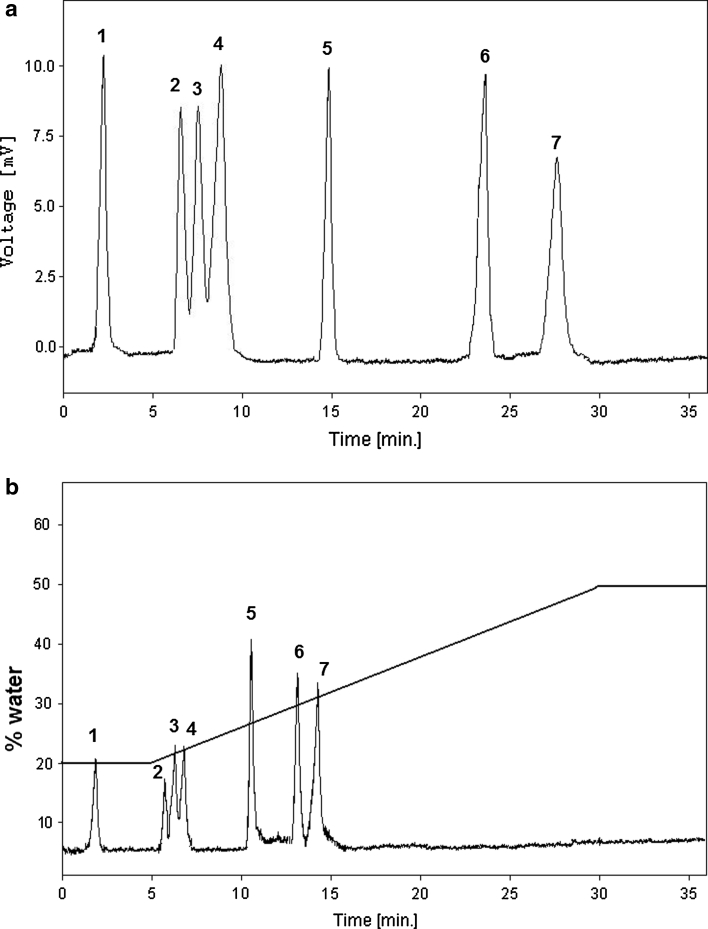
Separation of the vitamin B6 and six amino acids in **a** isocratic elution (composition of the mobile phase: 20 % (v/v) water, pH 3.0 (formic acid) + 80 % (v/v) ACN); **b** gradient elution (A: acetonitrile, B: water, pH 3.0 (formic acid), A/B (80:20) for 0 min to A/B (50:50) in 30 min, hold for 5 min, re-equilibrate A/B (80:20) for 5 min); for TSK-gel NH_2_ column. Flow rate: 1 mL min^−1^; ELSD detector: drift tube: 40 °C, nebulizer gas pressure: 2.3 bar, gain: 1. Notation: *1* vitamin B6, *2* tryptophan, *3* leucine, *4* isoleucine, *5* proline, *6* threonine, *7* glycine

As shown in Table 3, the separation efficiency of three systems for the seven sample compounds is higher in 20 % (v/v) water as the mobile phase than in 30 % (v/v) water. The sample compounds could not be separated in highly aqueous mobile phases under reversed-phase conditions. Figure 4a shows the complete separation of all selected amino acids and vitamin B6 in the mobile phase containing 20 % (v/v) of water. The retention time achieved was in <30 min. The analytes elute in order of increasing polarities. The analysis time can be reduced to 14 min using a gradient of increasing part of water concentration in the mobile phase (Fig. 4b).

### Concluding Remarks

Different stationary phases provide different separation selectivity and elution order. Mixed retention mechanisms were observed on TSK-gel Amide, TSK-gel NH_2_ and Luna HILIC column. A typical HILIC mechanism was observed at low water content (<40 %). The best results of seven biologically active compounds separation were obtained on the TSK-gel NH_2_ column. Amino-bonded phase showed mixed HILIC–ion-exchange mechanism, the highest separation efficiency and better selectivity and resolution for tested analytes than the other studied column, especially at concentration of water in mobile phase lower than 30 % (v/v). ELSD coupled with HILIC is a versatile and powerful technique to analyze amino acids and vitamin B6.
